# Cervical cerclage in advanced prolapsed fetal membranes - Case presentation 

**Published:** 2016

**Authors:** CF Tufan, RE Bohîlţea, A Tufan, A Marinescu, A Baros, MM Cîrstoiu

**Affiliations:** * "Carol Davila” University of Medicine and Pharmacy, Bucharest, Romania; ** Obstetrics and Gynecology Department, University Emergency Hospital Bucharest, Romania

**Keywords:** uterine cerclage, cervical insufficiency, cervical-length screening

## Abstract

The case presentation of a transvaginal cervical cerclage performed at a 7 cm dilation in a patient in the 22nd week of pregnancy, followed by a prolongation of the pregnancy until the gestational age of 38 weeks, was reported in the context of many similar cases managed by the authors of the article during a program of screening and prevention of preterm birth. The particularity of the case was the lack of a preterm birth in the medical history of the patient and the installation of the isthmus-cervix incompetence in the second pregnancy, after an on term pregnancy. What should be evidenced is the importance transvaginal cervical ultrasound evaluation has in the early diagnosis of this pathology during pregnancy, this being the only method of determining the efficacy of the content of the internal cervical os. Transvaginal cervical exploration has to be implemented as a screening method both in the high-risk patients and in the absence of a suggestive medical history.

## Case report

A 30-year-old, normoponderal, Arabian patient of Christian religion, established in Romania 8 years before, being at her second pregnancy 5 years after the first pregnancy, which evolved normally and ended with an on term vaginal pregnancy, was referred to the obstetrician at 5 weeks and 3 days of amenorrhea. The usual laboratory analyses were in normal ranges, the TORCH screening was negative for acute infections, the combined screening performed at 12 weeks of pregnancy, and the integrated one performed on demand at 16 weeks of pregnancy revealed low risks for chromosomes 13, 18 and 21 aneuploidies and for neural tube defects (NTDs). In the absence of risk factors, the cervical length was not measured transvaginally, as a screening method for the prediction of preterm birth.

**Fig. 1:**
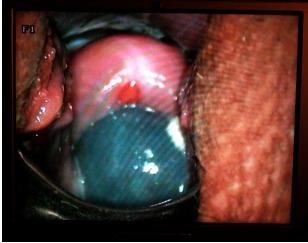
**Fig. 1**Indirect on screen image of the amniotic sac protruding through the cervical os into the vagina

At 22 weeks of pregnancy, the patient presented to the emergency with a sanguinolent leucorrhea in the absence of painful uterine contractions and, as epiphenomena, it only manifested with gingival pains. The clinical examination revealed a 7 cm dilation, and through the tension aminochorial membranes, the active movements of the fetal members were noticed, the prolapsed amniotic sac was in the vagina through the external cervical os (**Fig. 1**).

**Fig. 2:**
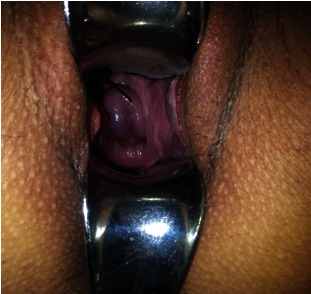
**Fig. 2**Direct image of the cervix immediately after applying the synthetic cerclage wire

After the ultrasound evaluation of the fetal viability and the gestational age, it was decided to perform the emergency cervical cerclage, with a facemask general anesthesia, progressively reducing the amniotic sac by traction on the edges of the cervix and a progressive basting of the number 2 nonabsorbable synthetic cerclage wire, through McDonald’s method, the patient being in Trendelenburg position (**Fig. 2**).

Placing a ring forceps or a stay suture of 00 silk around the circumference of the external os, represent alternative options. The gestational age less than 28 weeks allowed the rectal administration of Indometacin, a prostaglandin synthetase inhibitor, at the end of the procedure and in the next 48 hours after the cervical cerclage. The antibiotic prophylaxis was not supported by the recent negative cervical cultures. Moreover, the prophylaxis of the neonatal respiratory distress was performed with corticosteroids, according to the standard protocol; 6 mg of Dexamethasone were administered at 12 hours for 48 hours.

Under prophylactic treatment with natural progesterone microdose, intravaginally administered, and a transvaginal monitorization of the cervix at two weeks, the pregnancy presented a favorable evolution until the 38th week, when the extraction of the synthetic cerclage wire was followed by a spontaneous birth of an alive, male newborn child, weighing 3150 g, with an APGAR score of 9 at 1 minute from the birth and of 10 at 5 minutes after birth. 

The weight gain of the mother during the pregnancy was of 1340 g and the medium blood pressure was of 120/ 70 mmHg.

## Discussion

The case presented is part of a series of cases registered and analyzed by the authors, having as a common element the spontaneous and rapid installation of cervical insufficiency in patients with previous on term births, in the absence of other risk factors, without painful uterine contractions or prodromal symptoms of preterm birth. Among the patients whose cervical length was transvaginally evaluated at 12-13 weeks of gestational age, during the ultrasound examination of fetal anomalies in the first trimester and also at 16 weeks during the biometry necessary for the integrated test, none of them was enclosed in the high risk category of preterm birth, which brings into question the efficacy of universal screening, the gestational age which is optimum for the performance of screening and the duration of the negative predictive value confidence interval.

According to the guidelines of the Society for Maternal-Fetal Medicine, the definition of the short cervix, the estimation of the risk of preterm birth, and the therapeutic indication, are based on the medical history of preterm birth, the threshold value of transvaginal length of the cervix being of ≤ 20mm in patients without a case history who have to receive progesterone transvaginally and of < 25mm in patients with a positive case history who need cervical cerclage and administration of hydroxyprogesterone caproate [**1**,**2**].

The universal screening by transvaginal measurement of the cervix is not routinely recommended by the 2013 Cochrane review due to the lack of evidence [**3**,**4**] and it was not adopted at present by the specialty forums such as Society for Maternal-Fetal Medicine [**1**], American College of Obstetricians and Gynecologists (ACOG) [**5**,**6**], Society of Obstetricians and Gynaecologists of Canada (SOGC) [**7**], International Society of Ultrasound in Obstetrics and Gynecology (ISUOG) [**8**] and also not by the Romanian Society of Ultrasound in Obstetrics and Gynaecology (SRUOG). The algorithm of Berghella V. sustains the transvaginal measurement of the cervix between 14/16 - 24 weeks of pregnancy as a screening method in patients without a medical history of preterm birth and also as a follow up at 2 weeks and weekly in patients with a history of preterm birth and the cervix length of ≥ 30mm and < 30mm respectively. The performance of cervical cerclage at 12-14 weeks of pregnancy is indicated in patients with a medical history of preterm birth and cervix of < 25mm, and the administration of progesterone until the 36th week includes all the patients with a history of abortion in the second trimester or of spontaneous preterm birth, as well as the ones without a medical history but with the cervical length of ≤ 20mm [**9**]. In 2012, Cohrane’s review confirmed the efficacy of cervical cerclage, which, for single pregnancies significantly decreased the incidence of preterm birth [**10**]. However, the prolapsed fetal membranes through the external cervical os is considered a relative contraindication of the procedure due to the risk of iatrogenic rupture of the membranes, which is over 50% [**11**,**12**].

Our approach regarding the patients without risk factors refers to the transvaginal measurement of the cervix in standard conditions, at each prenatal consultation until 32 weeks of gestational age, when the ultrasound examination is performed in order to detect the fetal anomalies specific for the third trimester. Patients with spontaneous abortion in the second trimester, a history of preterm birth or risk factors for cervical insufficiency, as well as the ones diagnosed with short cervix, according to the previous definition, are recommended supplementary natural microdose progesterone intravaginally administered between 16 and 36 weeks of pregnancy and transvaginal follow up of the cervical length at 2 weeks until the 34th week of gestational age. The administration of vaginal progesterone to patients with a short cervix reduces the rate of spontaneous preterm birth, and improves neonatal morbidity and mortality rates and seems to be cost-effective. The patients with cervical length of < 17mm between 26 and 37 weeks of gestation have to be hospitalized in a 2nd or 3rd level unit in order to administrate acute tocolysis and corticosteroids prophylaxis, according to the standard recommendations. The reason for this attitude is based on the studies that prove the efficiency of progesterone addition and of cervical cerclage in lowering the incidence of preterm birth, on simplicity of transvaginal ultrasound measurement and on the good compliance of the patients. The performance of prophylactic cervical cerclage has the following indications: at least three 2nd trimester abortions or history of preterm births, two 2nd trimester abortions or history of preterm births for which no other cause but for cervical insufficiency could be identified, a 2nd trimester abortion or a preterm birth in the history of the pregnant patients in whom transvaginal ultrasound examination evidenced a shortage of the cervix to < 25mm accompanied by the modification of the internal cervix. The internal cervical os incompetence diagnosed by transvaginal ultrasound indicates the performance of cervical cerclage no matter the presence or absence of the risk factors [**13**], after the exclusion of pathogenicity of the cervical and vaginal bacterial flora, until the 28th week of gestational age. While being studied during an ultrasound examination, the anatomical and functional particularities of the internal cervical os need special attention. Our experience in the ultrasound evaluation of the cervix differentiates the linear shortage of the cervix between the two os contained by the shortage of the cervix through the “V” or “U” opening of the internal cervix. Taking into account that the opening of the cervical canal progress from the interior to the exterior, we consider that the purpose of the cervical cerclage is the restoration of the internal cervical continence when this cannot be accomplished anymore. Given the fact that the distance between the plane of the transversal cervical section, which contains the synthetic cerclage wire and the parallel plane which passes through the external cervical os usually does not exceed 2 cm, the invasive maneuver regarding the cervix, recommended by the Society for Maternal-Fetal Medicine for the treatment of the short cervix below 25 mm in patients with a history of preterm birth, is justified but only in case the internal cervical os cannot provide the contention.

Transvaginal cervical cerclage proved to be an efficient prevention method of preterm birth in the absence of painful uterine contractions, hemorrhage, ruptured membranes and of pathogenic colonization of the inferior genital tract, even when the membranes prolapse in the vagina through the internal cervical os, mostly when there is an early dilation and the abortion is impending and the small gestational age offers minimal survival rates and a high neonatal morbidity rate.

In addition, we suggest trying to keep the integrity of the aminochorial membranes; the benefit of the reduction of the intra-amniotic pressure through amniocentesis and the extraction of the amniotic fluid is counterbalanced by the fetal inflammatory response syndrome, which will form a progressive irritative sharp bony prominence during the further evolution of the pregnancy.

## Conclusions

The transvaginal measurement of the cervix is a simple, reliable method, well tolerated by the patients, highly sensitive and with a high positive predictive value in the process of evidencing the patients with a high risk of preterm birth, mostly in the presence of a positive medical history.

Although recent data are in favor of a lower incidence of short cervix in patients with a history of on term births compared to nulliparous patients [**14**], the presentation of the analyzed case favors the inclusion of this category of patients in the screening of preterm birth by transvaginal measurement of the cervical length.

The uterine cerclage performed at the moment of an advanced cervical dilatation represents a difficult trial of the obstetrician who has no other therapeutic option of approaching an imminent miscarriage. The success of the surgery in conditions of cervical insufficiency can offer an excellent maternal and fetal prognosis.
